# A Meta-Analysis Characterizing Stem-Like Gene Expression in the Suprachiasmatic Nucleus and Its Circadian Clock

**DOI:** 10.1155/2018/3610603

**Published:** 2018-06-26

**Authors:** Dilshan Harshajith Beligala, Arpan De, Michael Eric Geusz

**Affiliations:** Department of Biological Sciences, Bowling Green State University, Bowling Green, OH 43403, USA

## Abstract

Cells expressing proteins characteristic of stem cells and progenitor cells are present in the suprachiasmatic nucleus (SCN) of the adult mammalian hypothalamus. Any relationship between this distinctive feature and the master circadian clock of the SCN is unclear. Considering the lack of obvious neurogenesis in the adult SCN relative to the hippocampus and other structures that provide neurons and glia, it is possible that the SCN has partially differentiated cells that can provide neural circuit plasticity rather than ongoing neurogenesis. To test this possibility, available databases and publications were explored to identify highly expressed genes in the mouse SCN that also have known or suspected roles in cell differentiation, maintenance of stem-like states, or cell-cell interactions found in adult and embryonic stem cells and cancer stem cells. The SCN was found to have numerous genes associated with stem cell maintenance and increased motility from which we selected 25 of the most relevant genes. Over ninety percent of these stem-like genes were expressed at higher levels in the SCN than in other brain areas. Further analysis of this gene set could provide a greater understanding of how adjustments in cell contacts alter period and phase relationships of circadian rhythms. Circadian timing and its role in cancer, sleep, and metabolic disorders are likely influenced by genes selected in this study.

## 1. Introduction

 Circadian rhythms that control daily behavior and physiology of mammals are regulated by a timing system in which multiple circadian clocks in the organs and tissues interact with a master clock in the suprachiasmatic nucleus (SCN) of the hypothalamus [1–3]. The SCN is a relatively small brain area positioned just beyond the optic chiasm where it receives signals directly from the retina. These light signals and additional synaptic and hormonal inputs entrain the SCN's clock so that the circadian system remains synchronized to predictable daily events in the environment.

Along with its role in processing light signals and generating circadian rhythms, the SCN has an additional distinctive feature that has not yet been explained. Many of its cells express an unusual number of genes that would be expected in fetal and early postnatal brains but not in mature brain tissue other than the few areas in which elevated ongoing and induced adult neurogenesis occurs. For example, Sox2 is a common cell-specific marker for the stem cell state [4] and is also expressed in the adult SCN [5]. Ube3a gene expression colocalizes with Sox2 expression in the adult SCN. When mutated it causes neural developmental disorders and sleep disruption, possibly through its actions on core clock proteins [6], which could indicate a role for SOX2 in the adult SCN by association. Doublecortin (DCX) and doublecortin-like (DCL) proteins are usually found in neuroblasts undergoing a final differentiation into neurons and radial glial cells, but their genes are also expressed in the adult SCN [7, 8]. Several of these neurogenesis-related genes regulate each other. For example, virus-driven SOX2 expression induces DCX-positive neuroblasts, and induced pluripotent stem cells made from astrocytes show a sequence of differentiation from SOX2 through DCX expression [9]. Six3 is expressed in developing brain and its loss prevents SCN formation, yet it is also expressed prominently in adult SCN cells [10]. Furthermore, the SCN's unusually low expression of NeuN (Rbfox3) [7], a marker for mature neurons, also suggests that many SCN neurons may not be in a fully differentiated state. Nevertheless, SCN neurons are adequately mature to generate spontaneous action potentials in robust circadian rhythms [11].

A puzzling aspect of these stem-like features is that the adult SCN shows conspicuous expression of stem cell marker proteins but lacks obvious neurogenesis [12]. Because most SCN histological studies have relied on animals maintained under highly regulated laboratory and animal care conditions it is possible that the SCN has a neurogenesis program that is initiated more often in animals experiencing their natural environment and in response to episodic stressors and challenges throughout the lifetime [13]. Here, we provide evidence that the SCN's unique stem-like state reflects immature cells that retain a degree of plasticity allowing them to adaptively rearrange neuronal circuitry responsible for modifying the SCN's circadian rhythms. Several researchers have reported that cell-cell contact, the extracellular matrix, and synaptic plasticity alter the SCN circadian clock's period and entrainment [14–16], and the circadian clock can in turn regulate synaptic strength [17]. We also examine here the possibility of a latent feature of SCN cells to undergo episodic adult neurogenesis when appropriate conditions arise.

The SCN network of circadian clock cells is a heterogeneous population of neurons and glial cells. There has been substantial progress in explaining the intracellular timing mechanism within individual SCN clock cells, but mathematical modeling is needed to understand how the ensemble output of multiple SCN neurons determines the pattern of circadian timing information reaching the rest of the brain and body [18]. Several network models of coupled SCN clock cells include flexibility of neuron interactions and their responses to external demands on the animal [19–22]. Examining all of the most probable circuit modulators is needed to understand how the collective rhythmic pattern originates within the SCN cell network. Clearly, GABA neurosecretion is important in forming the ensemble circadian rhythm along with synaptic transmission within and between large populations of neurons producing vasoactive intestinal polypeptide (VIP), arginine vasopressin (AVP), gastrin releasing peptide (GRP), and calretinin (CR) or calbindin [18, 23–26]. Nevertheless, several other nonneuronal cell types play important but poorly defined roles [27, 28]. Astrocytes interdigitate between SCN neurons, likely modulating synaptic strength, and display circadian rhythms in expression of the astrocyte marker glial fibrillary acidic protein (GFAP) [29]. Consequently, a much clearer understanding of how the SCN adjusts its circuitry to modulate rhythmic output requires a broader examination looking beyond neurons and encompassing cell interactions of intrinsically rhythmic and nonrhythmic network components [30].

The SCN timing signals that essentially schedule appropriate animal behavior relative to local time originate mostly within a population of SCN neurons that are most notable for their arrangement in a dorsomedial “shell” area surrounding a distinct ventrolateral “core” of cells [1]. Although numerous neuropeptides are expressed within the SCN, the shell is notably dominated by neurons expressing AVP, whereas cells in the core express GRP and conspicuous levels of VIP, which is found in few other brain areas. Substantial evidence supports retinal axons projecting principally to the core, but also to other SCN cells. VIP receptors of shell neurons respond to core neurons, and shell AVP neurons communicate with VIP neurons as well as each other [16, 31]. These connections provide tight coupling of the circadian clocks within cells receiving these timing signals and possibly others more indirectly connected through cell interactions not yet well understood. Furthermore, several studies have supported a plasticity in the phase relationships between the shell and core SCN, particularly while the SCN clock responds to light exposure by either advancing or delaying its phase [11].

Otherwise, the SCN seems to lack organized arrangements of synapses or cell bodies like those of cortical areas, cerebellum, olfactory bulb, etc. in which cell layers and fiber tracts are understood to convey specific information relevant to each brain substructure. Although clustering of locally interacting SCN neurons of specific phenotypes has been described [16], any rigidly defined cell connectivity remains elusive, despite patterns of rhythmic neural activity and gene expression indicating information passing between areas [32]. Although not conclusive, this apparently looser SCN architecture raises the possibility of cell motility and rearrangement of cell contacts. Several studies have examined SCN expression of proteins considered important in cell-cell communication other than through neurosecretion [33], and the evidence indicates they alter the period of ensemble SCN rhythms and responses to retinal light signals. Adjustments in SCN cell interactions modify rhythmic behavior and may be needed adaptively to survive changing seasonal conditions or altered food availability.

This study was initiated to provide a more comprehensive assessment of stem cell marker proteins and related cell contact proteins in the mouse SCN based on published reports and databases describing the SCN transcriptome. Cells that are partially differentiated or have undergone dedifferentiation, as cancer cells do prior to metastasis, are known to express a distinct set of proteins indicating loss of endothelial cell connectivity and greater interaction with the extracellular matrix and other cell types. If modifying cell-cell contacts is important for basic SCN timing functions, then it can be predicted that SCN cells do express a substantial number of the known proteins supporting these connections and their maintenance, including proteins conspicuous in cancer. Results presented here describe several genes of these poorly differentiated cell types that are expressed in the SCN and are promising candidates for additional exploration of circadian network properties.

## 2. Materials and Methods

### 2.1. Databases Queried

Descriptions of major functions of mouse genes and their human orthologs were derived primarily from the following databases: GeneCards (http://www.genecards.org/), Gene Ontology (GO) Consortium (http://www.geneontology.org/) [34], PANTHER (http://pantherdb.org/) [35], and Entrez from the National Center for Biotechnology Information (https://www.ncbi.nlm.nih.gov/) [36]. Images showing in situ hybridization (ISH) results in sections of the adult mouse brain were obtained from the Allen Institute Mouse Brain Atlas (ABA) and are available online at http://mouse.brain-map.org/ [37]

### 2.2. Procedure for Selecting Candidate Stem Cell-Related Genes

To select for candidate genes with a role in stem cells and related cell-cell interaction the genes listed in the Fine Structure Search (FSS) of the ABA were examined. The FSS provides a curated list of genes expressed at high levels in a user selected brain area and ranks the genes based on their expression level and whether they are predominately expressed in that location when compared with other brain areas. A set of genes producing gene regulators reported to have enriched expression in the mouse adult SCN by Hatori et al., 2014 [38], was also examined. These genes and the SCN FSS genes were evaluated further based on a search of the PANTHER database, where Complete GO Bioprocess (BP) listings were explored. BP terms relevant to development, differentiation, stem cell maintenance, cell-cell contact, or contact with extracellular matrix related to stem cells were gathered. Any terms relevant to the nervous system were selected first. Some related bioprocesses relevant to these stem cell target areas were not included in the resulting list because of space limitations. The candidate genes selected because of their GO BP attributes were tested further using the Differential Search feature of the ABA to determine their expression level in the SCN versus several brain areas.

The same genes were also examined in CircaDB and SCNseq online databases. CircaDB provides the expression of mouse and human genes at time intervals throughout the circadian cycle and also enables statistical analyses (http://circadb.hogeneschlab.org/) [39]. The SCNseq database provides gene expression data over a 24-hour cycle from SCN of mice maintained in cycles of 12 hours of light and 12 hours of dark, thereby including additional light-driven gene expression (http://www.wgpembroke.com/shiny/SCNseq/) [40].

Finally, the selected candidate genes and additional relevant genes of interest were evaluated through the Differential Search feature of the ABA that provided the fold change in expression between the SCN (target area) and various contrast areas.

## 3. Results and Discussion

### 3.1. FSS Analysis

Genes listed in the FSS, which are principally expressed in the SCN and at high levels, were screened for any described role in development, differentiation, stem cell maintenance, cell-cell contact, or contact with extracellular matrix related to stem cells. The list includes obvious intrinsic cell membrane proteins and not only denizens of the extracellular space, but also intracellular cell signaling molecules mediating cell-cell contact information. Of the 47 genes in the FSS, 17 were found to have a role in stem cells or closely related processes (Table 1).

Genes with only a role in chemical or electrical synaptic communication were not included. Nevertheless, the targeted biological processes were well represented in the SCN transcriptome relative to other hypothalamic structures (Table 1). For some of the genes the lack of evidence showing involvement in stem-like properties may be because of few reported studies describing their functions.

Any mentioned role in circadian rhythms was included in Table 1, and the phase of any of the genes reported to display a significant circadian rhythm in expression was included, as shown by the phase of the oscillation's peak as reported in CircaDB. Furthermore, the phase of the peak expression in mice maintained in a light/dark cycle was included along with an indication of whether the oscillation had a statistically significant fluctuation according to SCNseq results.

### 3.2. Evaluating Transcription Factors Expressed at High Levels in the SCN

As an additional evaluation of the stem-like properties of the SCN we examined the gene expression results of Hatori et al. (2014), who identified 13 transcription regulator genes that have elevated expression in the SCN [38]. Interestingly, nine of these enriched genes are involved in stem cells or development according to gene annotations in PANTHER (Table 2), and only two of the 13 genes did not show a relevant gene association—Gatad2b (GATA zinc finger domain containing 2B) and Hsf2 (heat shock factor 2). As in Table 1, any gene showing significant circadian rhythms was indicated by the phase of the rhythm's peak, and the peak expression in mice maintained in a light/dark cycle was included along with indication of any significant amplitude.

Because it was not a focus of this study, BP annotations for gene regulation were not typically included. Some of the genes (Dlx2, Lhx1, Nr2f2, Rora, Six3, Sox1, Sox11) have a large number of BP annotations for stem cell processes including differentiation and stem cell maintenance outside the nervous system, which were not listed here. Some of the reports of stem-like properties include artificial manipulations outside embryonic or fetal development such as the ability of Foxd1 to reprogram induced pluripotent stem cells [41].

### 3.3. Genes Expressed in the SCN That Serve in Stem Cell-Regulating Pathways

Table 3 provides additional evidence that the genes in Tables 1 and 2 are expressed at higher levels in the SCN relative to other brain areas and so are likely to have a function relevant to SCN properties including the circadian clock. Genes in Tables 1 and 2 were explored further through the Differential Search feature of the ABA with the SCN serving as the target structure and various other brain areas serving as the contrast structure. The fold expression shown is the target gene expression divided by the contrast structure expression. If expression was considered by the routine to be absent in either structure, no value was returned. Such was the case for Lhx1, which was expressed almost entirely in the SCN. Only coronal data were included to avoid comparisons across different SCN subregions. Many of the genes in Table 2 that were not in Table 1 did not generate a result because they either were missing from the ABA (Sox1) or were not included in experiments using coronal sections (Dlx6, Foxd1, Nr2f2, Six3, Sox1, Sox11) or were otherwise unavailable (Dlx2).

It was clear from the Differential Search results that the candidate genes are expressed at higher levels in the SCN than the hypothalamic nuclei examined. As listed in Table 3, large percentages of the candidate genes were expressed in the SCN relative to the contrast areas. Both the paraventricular hypothalamic nucleus (PVH), which receives SCN neuronal projections, and the PVH descending division (PVHd) showed far lower expression of nearly all of the candidate genes. Expression of nearly all of these genes was greater in the SCN than the arcuate nucleus of the hypothalamus (ARH), a structure with reported adult neurogenic abilities [13, 42]. The percentage of genes expressed at a higher level in the SCN declined when it was compared with the dentate gyrus (DG, 63%), which was not surprising considering the large number of stem cell-related genes expressed in this well-established site for rodent adult neurogenesis. Nevertheless, the replicate experiments provided in the ABA for some of the candidate genes in Table 3 showed a large range of fold change indicating that this approach is effective for comparing two structures but is at best semiquantitative.

Interestingly, all but five of the genes in Table 3 (Dlk1, Lhx1, Pcsk2, Rora, Rorb) were visibly expressed in the rostral migratory stream structure that delivers neuroblasts to the olfactory bulb where they differentiate into interneurons [43]. This observation provides additional evidence that many of the candidate genes serve a function related to neurogenesis. It is possible that the mobile or altered cell interaction properties of neuroblasts are maintained in SCN cells by these and related genes, whether or not they fully differentiate into mature neurons.

Tables 1 and 2 also provide evidence that stem cell-regulating pathways are involved in maintaining SCN stemness. For example, Nkd1, Six3, and Tle4 expression serves in Wnt signaling. Interestingly, stem cell replication in the mouse intestine is controlled by circadian rhythms in WNT secretion from Paneth cells [44]. The gene annotations also showed that expression of Dlk1 and Zic1, which along with Rora regulate smoothened homolog (Smo), is linked to hedgehog pathways [45]. The ABA reveals moderate expression of Sonic hedgehog (Shh), Indian hedgehog (Ihh), and Desert hedgehog (Dhh) in the SCN based on visual inspection of ISH in brain sections.

Genes coding for Notch proteins were expressed weakly in the SCN according to the intensity of ISH signals in the ABA. Nevertheless, there was evidence for noncanonical Notch expression. For example, Dner (Delta/Notch-like EGF repeat containing) is highly expressed in the SCN, suggesting that it may have a role in processes relevant to the SCN such as responses to photic sensory signals or production and modulation of circadian rhythms. Dner is expressed at relatively lower levels in some but not all neighboring brain areas. A Differential Search in the ABA found 17.75, 0.824, and 0.577-fold expression in the SCN when compared with the SO, LPO, and PVH, respectively. It was expressed only 0.702-fold in the SCN relative to the DG.

Similarly, strawberry notch homolog 1 (Sbno1) was expressed moderately in the SCN. A Differential Search found that Sbno1 is expressed at higher levels in the SCN than the PVH and SO (2.495- and 7.166-fold, respectively) and only 0.288-fold when compared with the DG, most likely because of its possible role in DG neurogenesis. Members of a gene family expressing important regulators of Notch signaling (Adam10, 11, 15, and 23) showed moderately high expression in the SCN and other hypothalamic nuclei following visual inspection. Thus, many of the components related to the Delta/Notch signaling pathway are induced in the SCN. Additional studies should determine which of these genes act together in a functional pathway to regulate stem-like cells and possibly suppress differentiation.

Additional genes in the FSS list that should be examined for possible roles in altering SCN cell interactions are Epha6 (Eph receptor A6) and Blcap (bladder cancer associated protein). EPHA6 protein maintains communication between adjacent cells, and Epha6 expression is under circadian control in the SCN according to CircaDB. Blcap counters cell proliferation by increasing apoptosis. These two genes along with Zfhx3 and several others in the FSS are also involved in cancer cell activity [46] and others, such as Btg1 and Dlk1 control cell division, a process more relevant to a tumor than a neural structure not currently considered a site of cell renewal.

Why there is a pattern of gene expression in the SCN that overlaps genes expressed in stem cells and cancer cells remains unclear other than the generally undifferentiated state of cancer cells and cancer stem cells, in particular [47]. To dissect this pattern more finely, studies could differentiate between stem-like properties of the SCN that are more closely associated with cancer stem cells and those of noncancerous embryonic or adult stem cells. Similarly, selecting genes showing preferred expression in gliomas rather than glial cells could further define the pool of stem-like genes expressed in the SCN. The resulting set of potentially interacting genes might reveal what functions this population serves.

### 3.4. Stem Cell-Related Genes Regulating SCN Circadian Rhythm Output or Phase Shifts through Cell-Cell Coupling

It is well established that manipulation of genes expressing core proteins of the circadian clock timing mechanism or genes that regulate these proteins can alter or even eliminate the circadian rhythm's period within the SCN [48]. Substantial research has shown that circadian clocks of organisms depend on individual cells endowed with these intrinsic circadian timing abilities. It is commonly stated that circadian clocks are present within nearly all cells of the body, but some cells and tissues lack an ability to sustain normal circadian rhythm generation without close proximity to other cells [49] or intermittent synchronizing timing signals from more robust and sustained clocks such as the one in the SCN [50]. Many cell lines, for example, will show their abilities to express a circadian rhythm after synchronization of their circadian oscillators with agents that induce cell signaling pathways and gene expression such as dexamethasone, forskolin, or a “serum shock” in which a high level of serum is delivered transiently to cells previously deprived of serum [51–53]. These “resetting” methods bring the individual circadian clocks, which presumably are within nearly all the culture's cells, to a common phase of the cycle. This synchronization activates genes serving within the central circadian timing mechanism and is much like the ability of strong photic stimuli to reset circadian clocks within animals or animal populations to a common circadian phase.

Circadian rhythms have been recorded in cell lines without using resetting procedures, which suggests there is either spontaneous synchronization through intercellular communication or an unintended delivery of a timing signal (Zeitgeber) such as the stimulation imposed by culture medium exchange [53, 54]. Circadian rhythms recorded in cell cultures, tumor spheroid cultures, and tissue explant cultures in the absence of a specific synchronizing treatment suggest that cell-cell communication can bring circadian clocks within cells of a culture into a common phase and maintain them in a coherent rhythm [11, 23].

The SCN in brain slices maintained as explant cultures expresses many cycles of circadian activity without need for a synchronizing agent, providing further evidence of the tight coupling between its cellular clocks. Nevertheless, that coupling can be weakened, resulting in a reduced rhythm amplitude and loss of circadian behavioral activity rhythms controlled by the SCN. This influence on cell-cell coupling was reported by knocking out the Lhx1 gene in mice, which appears to regulate cell interactions through VIP and possible SCN cell interactions with the extracellular matrix [38, 55]. Lhx1 expression in the SCN is also suppressed by retinal light exposure [38]. Lhx1 has emerged as an important potential regulator of cell coupling within the SCN with effects on period and phase shifts. Evidence provided here suggests that it is among a group of genes that function in maintaining the stem-like state of cells and associated alterations in cell shape and motility.

Stem cells and migrating cancer cells show altered cell-cell interactions because of a switch in their gene expression patterns, producing distinct morphological alterations including the epithelial-to-mesenchymal transition (EMT) of migrating or metastatic cancer cells [47, 56]. Proteins that serve in cell-cell interactions also modify the SCN's rhythmic output, supporting the idea that the stem cell-like state of SCN cells functions in providing plasticity of circadian timing. In addition to Lhx1, Zfhx3 expression is reported to regulate coupling between SCN neurons [57]. Lhx1 serves in SCN development and when it is knocked out in the SCN the rhythmic output is disrupted, circadian locomotor activity is diminished, and peptidergic neurons (VIP, AVP) that provide rhythmic output are fewer [38, 55, 58]. When the transcription factor ZFHX3, which functions in neurulation and neuronal terminal differentiation, is knocked out in the adult SCN the wheel-running rhythm in constant darkness is shortened by over an hour or becomes completely arrhythmic [59].

One example of a gene family that is altered substantially during EMT is the cadherins, and interestingly Cdh13 (cadherin 13) is listed in the FSS because of its high expression in the SCN. Another Table 1 gene that invites further scrutiny for its possible role in SCN rhythm modification is Flrt3 (fibronectin leucine rich transmembrane protein 3), which has many roles in cell-cell contact control and synapse plasticity. This gene should be examined further because of the SCN's major glutamatergic retinal afferents and the role of FLRT3 in altering glutamate synapse development [60]. One question is whether FLRT3 maintains or modifies SCN afferents in the adult. Evidence indicates that interfering with the polysialylated derivative of neural cell adhesion molecule (NCAM), which alters cell-cell contacts and glutamatergic synapses, prevents retinal light signals and other synchronizing stimuli from shifting the phase of the SCN clock [14].

### 3.5. Summary of Candidate Stem-Like Gene Activity in Adult Mouse SCN

One additional question is whether some of the candidate stem cell genes are expressed in the same cells, where their products may interact. We observed in the ABA that the candidate genes were expressed in cells with different morphologies and distributions in the SCN, ranging from rounded neuron-like cells to much smaller cells and cells resembling astrocytes. Nevertheless, the candidate genes could serve in coupling between cell types within networks that include the established interactions through VIP and AVP, providing additional modifications of SCN rhythms and phase shifts in response to light.

Potentially, the in situ gene expression data examined in Tables 1 and 3 included light-induced expression as well. We can presume that the mice used for the ABA were euthanized during exposure to visible light, and probably this was during the animal's daytime. Therefore, it should be considered that all of the expression values shown in the tables could represent, to a variable extent, the light-induced state of the genes. In Tables 1 and 2, we included an estimate of the responsiveness of the candidate genes to light from data in SCNseq. A more thorough examination of light-dependent responses of the genes in Table 2 can be found in Hatori et al., 2014 [38].

**Figure 1:**
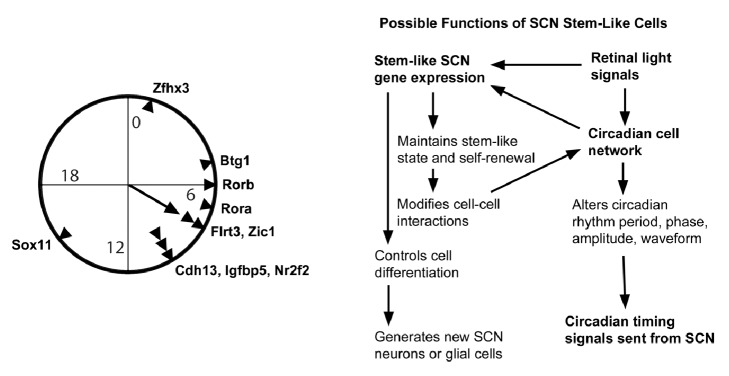
**Summary of possible interactions between the SCN circadian clock and genes associated with stem cells and neurogenesis. Left:** circadian phases of SCN stem cell-related genes. Phases of maximal expression of rhythmic genes from Tables 1 and 2 are shown (arrowheads). Significant clustering was around 7:59 as shown by the mean phase vector (arrow). The time indicated is relative to the prior light/dark cycle of the animals in which hour zero equals the time when light onset would have occurred and dusk would have been at hour 12.** Right:** theoretical functions of stem-like genes in the SCN. Entraining light signals act on circadian clocks within cells of the SCN cell network and also induce stem cell-related genes. Stem cell properties include altered cell interactions, providing a plasticity in cell networks that ultimately changes the generated circadian rhythm. Neurogenesis in the adult SCN remains a possibility but requires additional supportive evidence.

The phases of gene expression in Table 1 that were significantly rhythmic in the SCN of mice maintained in darkness are presented in Figure 1. Significant clustering of phases was detected by Rayleigh test (Z=4.22, p=0.011) and Rao test (U=177, p< 0.05). Six of the genes in Table 1 have highest expression near the time of maximal expression of core clock genes Rora and Rorb (6:00-7:00), suggesting that they are under similar circadian control. Lhx1 also shows maximal expression at 6:00 and has an estimated 32-hour period, just outside the circadian range considered here. Because Rora and Rorb are induced by BMAL1 and CLOCK it is likely that many of the other candidate stem cell genes are regulated through this same transcription factor complex or indirectly by this clock output pathway. It is also possible that the SCN's circadian rhythm in intracellular Ca^2+^ levels or the elevated neuronal firing rate preceding this phase induces expression of some of these genes. Whatever function the stem-like state of the SCN provides may be explored best by addressing why these clock-controlled genes are most active during this portion of the cycle. It has been suggested that SCN cells of mice and perhaps other night-active animals reorganize their connections during the night and that gene expression during the day is in preparation for the circuit plasticity that follows [38]. This possibility might explain the phase of candidate gene expression maxima during the day.

## 4. Conclusions

These results indicate that many genes expressed most prominently in the SCN are ones known or suspected to serve in maintenance of stem cell states and stages of neurogenesis and neural circuit formation. These processes include control of stem cell and progenitor cell proliferation and differentiation as well as later events including axon lengthening and synapse formation of maturing neurons. Furthermore, 10 of these 25 candidate genes are under circadian clock control and most are expressed in the SCN at highest levels during mid-to-late daytime. In fact, two of the genes are known components of the core circadian clock mechanism that also serve in development. Of the genes controlling the status of stem cells, expression data indicate that Wnt and noncanonical Notch pathways are likely to have important roles in the SCN. One possibility is that SCN cells upregulate proteins providing increased plasticity in their neural circuits by inducing gene regulatory pathways of stem cells. This adaptive flexibility may be required to allow the circadian clock to match timing system functions to changing environmental variables delivered in part to the SCN by retinal signals.

## Figures and Tables

**Table 1:** Genes expressed in adult mouse SCN selected from the Fine Structure Search.

Gene symbol and name	PANTHER GO Biological Process (GO BP Complete)	FSS ranking	Circadian phase in SCN (hours)	LD phase in SCN (hours)	Fluctuating in LD
**Lhx1 **(LIM homeobox protein 1)	nervous system development, cell-cell signaling	2	ND^1^	6	No^2^
**Rorb** (RAR-related orphan receptor beta)^3^	regulation of circadian rhythm, amacrine cell differentiation, retina development in camera-type eye, negative regulation of osteoblast differentiation	6	6	0	Yes
**Rora** (RAR-related orphan receptor alpha)^3^	regulation of circadian rhythm, regulation of smoothened signaling pathway, cerebellar Purkinje cell differentiation, negative regulation of fat cell differentiation	10	(7)	6	Yes
Flrt3 (fibronectin leucine rich transmembrane protein 3)	neuron projection development, synapse organization, fibroblast growth factor receptor signaling pathway, axon guidance, cell adhesion, response to axon injury, synapse assembly, embryonic morphogenesis, neuron projection extension, cell-cell adhesion via plasma-membrane adhesion molecules, positive regulation of synapse assembly	13	8	0	Yes
Zic1 (zinc finger protein of the cerebellum 1)	nervous system development, regulation of smoothened signaling pathway, spinal cord development, adult walking behavior, central nervous system development, cell differentiation	14	(8)	5	No
Btg1 (B cell translocation gene 1, anti-proliferative)	positive regulation of myoblast differentiation, positive regulation of endothelial cell differentiation	16	(5)	8	Yes
Spon1 (spondin 1, extracellular matrix protein)	cell adhesion	19	ND	9	No
Dlk1 (delta-like1 homolog Drosophila)	post-embryonic development, negative regulation of fat cell differentiation, negative regulation of Notch signaling pathway, osteoblast differentiation	21	ND	0	Yes
Neurod2 (neurogenic differentiation2)^4^	regulation of synapse maturation, nervous system development, positive regulation of neuron differentiation, regulation of neuron differentiation, associative learning, cerebellar cortex development, behavioral fear response, positive regulation of synaptic plasticity, cell differentiation, cellular response to electrical stimulus	22	ND	13	Yes
Igfbp5 (insulin-like growth factor binding protein 5)	negative regulation of muscle tissue development, negative regulation of osteoblast differentiation, striated muscle cell differentiation, osteoblast differentiation, negative regulation of cell migration	24	(10)	6	Yes
Thsd7b (thrombospondin, type I, domain containing 7B)	anatomical structure morphogenesis, ectoderm development^5^	26	ND	22	Yes
Msi2 (musashi RNA-binding protein 2)^6^	stem cell development	32	ND	8	No
Nkd1 (naked cuticle 1 homolog Drosophila)	positive and negative regulation of canonical Wnt signaling pathway, spermatogenesis, cell differentiation	34	ND	2	No
Myt1 (myelin transcription factor 1)	post-embryonic development, nervous system development, cell differentiation, endocrine pancreas development	35	ND	20	No
Zfhx3 (zinc finger homeobox 3)	nervous system development, cerebellar Purkinje cell differentiation, embryonic retina morphogenesis in camera-type eye, cell-cell signaling	36	1	6	No
Cdh13 (cadherin 13)	calcium-dependent cell-cell adhesion via plasma membrane cell adhesion molecules, endothelial cell migration, positive regulation of cell-matrix adhesion, positive regulation of cell migration	46	10	20	No
Pcsk2 (proprotein convertase subtilisin/kexin type 2)	nervous system development	49	ND	13	No

Boldface indicates a gene also present in Table 2. ^1^Lhx1 expression shows a 32-hour rhythm in the mouse SCN by JTK analysis (CircaDB). Acceptable circadian periods in this study were 19-30 hours. ^2^Lhx1 is suppressed by light [38]. ^3^Circadian clock-related or core clock gene. ^4^Neuronal differentiation marker. ^5^Only the GO slim annotation was available. ^6^Stem cell marker. ND = no significant rhythm detected in CircaDB using the JTK test. () = average phase when phases from multiple experiments with circadian rhythms in the SCN were reported in CircaDB.

**Table 2:** SCN-enriched transcription regulator genes identified by Hatori et al., 2014 [38].

Gene symbol and name	PANTHER GO Biological Process (GO BP Complete)	Circadian phase (hours)	LD phase (hours)	Fluctuating in LD
Dlx2 (distal-less homeobox 2)	cerebral cortex GABAergic interneuron differentiation and fate commitment, negative regulation of Notch signaling pathway	ND	8	No
Dlx6 (distal-less homeobox 6)	inner ear morphogenesis, epithelial cell differentiation, positive regulation of epithelial cell proliferation	ND	8	Yes
Foxd1 (forkhead box D1)	axon guidance, positive regulation of kidney development, positive regulation of bone morphogenetic protein signaling	ND	6	No
**Lhx1** (LIM homeobox protein 1)	nervous system development, cerebellar Purkinje cell differentiation, cell-cell signaling, embryonic retina morphogenesis in camera-type eye	ND	6	No
Nr2f2 (nuclear receptor subfamily 2, group F, member 2)	neuron migration, forebrain development, anterior/posterior pattern specification	10	6	Yes
**Rora (**RAR-related orphan receptor alpha)	cerebellar granule cell precursor proliferation, cerebellar Purkinje cell differentiation, circadian regulation of gene expression, cellular response to tumor necrosis factor, regulation of smoothened signaling pathway, negative regulation of fat cell differentiation, muscle cell differentiation, T-helper 17 cell differentiation	(7)	6	Yes
**Rorb **(RAR-related orphan receptor beta)	amacrine cell differentiation, retina development in camera-type eye, regulation of circadian rhythm	6	0	Yes
Six3 (sine oculis-related homeobox 3)	negative regulation of neuron differentiation, circadian behavior, neuroblast differentiation and migration, negative regulation of Wnt signaling pathway	ND	6	Yes
Sox1 (SRY (sex determining region Y)-box 1)	neuron migration, forebrain neuron differentiation, nervous system, development	ND	6	Yes
Sox11 (SRY (sex determining region Y)-box 11)	positive regulation of neuron differentiation, glial cell development, positive regulation of neurogenesis, positive regulation of hippo signaling, positive regulation of stem cell proliferation	(15.5)	6	No
Tle4 (transducin-like enhancer of split 4)	Wnt signaling pathway	ND	2	Yes

Boldface indicates genes also listed in Table 1. Circadian phases are from CircaDB. ND = no significant rhythm detected in CircaDB using the JTK test. () = average phase when phases from more than one experiment were reported for a rhythm in the SCN. Acceptable circadian periods were between 19-30 hours. Phases in light/dark cycle (LD) and significance of daily fluctuations are from SCNseq.

**Table 3:** Fold change in gene expression in the SCN relative to contrast areas examined through Differential Search analysis.

Gene symbol	Fold change in the SCN compared to contrast region						
	SO	LPO	PVH	PVHd	ARH		DG
Btg1	9.097	4.689	1.579	4.099	**0.339**		**0.231**
Cdh13	3.554-6.873	1.585-2.852	2.704	1.033-3.584	1.291-23.019		2.509-4.947
Dlk1	4.535	4.479	1.628	1.32	1.067		123.262
Flrt3	25.477	2.969	14.839	5.19	3.383		**0.721**
Igfbp5	10.391	7.541	3.416	2.868	2.321		1.34
Lhx1	NE	NE	NE	NE	NE		NE
Msi2	5.175	2.019	2.333	10.993	2.456		3.7
Myt1	3.055	2.825	1.323	1.08	1.062		**0.944**
Neurod2	3.517	4.373	2.807	NA	3.845		**0.855**
Nkd1	1.964	5.242	**0.88**	**0.7**	2.079		2.685
Nr2f2	2.292	4.411	NE	4.844	1.476		2.362
Pcsk2	3.872	NE	**0.941**	1.028	1.506		**0.944**
Rora	16.865-29.842	22.593-22.99	3.44-9.991	1.919-6.545	16.377-18.926		8.694-11.126
Rorb	35.538	19.534	8.161	18.822	9.207		32.208
Spon1	1.629	1.865	1.562	1.076	**0.82**		**0.612**
Thsd7b	4.586	**0.755**	**0.566**	NE	4.101		1.595
Tle4	40.204	13.117	NE	20.09	3.658		**0.915**
Zfhx3	5.525	3.685	2.379	1.803	1.659		9.041
Zic1	2.47	1.839	**0.639**	NE		29.025	2.416

% of genes with higher SCN expression	100	94.7	78.9	94.4		89.5	63.2

Boldface indicates where the contrast area had higher expression. The range of fold change is shown when more than one experiment was available for comparison. The maximum number of experiments per gene was three or less. NE: no significant expression in the contrast area. NA: target area expression was not available. SO: supraoptic nucleus, LPO: lateral preoptic area, PVH: paraventricular hypothalamic nucleus, PVHd: PVH descending division, ARH: arcuate nucleus of the hypothalamus, and DG: dentate gyrus.

## Data Availability

Data will be made available following a request to the corresponding author. Databases and other online resources used in this study are listed in the Materials and Methods section.
